# Editorial: The combination of data-driven machine learning approaches and prior knowledge for robust medical image processing and analysis

**DOI:** 10.3389/fmed.2024.1434686

**Published:** 2024-05-31

**Authors:** Diwei Zhou, Jinming Duan, Chen Qin, Gongning Luo

**Affiliations:** ^1^Department of Mathematical Sciences, School of Science, Loughborough University, Loughborough, United Kingdom; ^2^School of Computer Science, University of Birmingham, Birmingham, United Kingdom; ^3^Department of Electrical and Electronic Engineering & I-X, Imperial College London, London, United Kingdom; ^4^Faculty of Computing, Harbin Institute of Technology, Harbin, Heilongjiang, China

**Keywords:** deep learning, medical imaging, diagnostic accuracy, data-driven approaches, prior knowledge, robustness

Combining data-driven machine learning with prior knowledge has significantly advanced medical image processing and analysis. Deep learning, driven by large datasets and powerful GPUs, excels in tasks like image reconstruction, segmentation, and disease classification. However, these models face challenges such as high resource demands, limited generalization, and lack of interpretability. In contrast, model-driven approaches offer better generalization, interpretability, and robustness but may lack accuracy and efficiency. Combining these paradigms leverages their strengths, promising superior performance and enhanced diagnostic accuracy. This Research Topic showcases how this integration enhances medical imaging, including accurate stroke onset estimation, improved COVID-19 diagnosis and recovery assessment, and enhanced cardiac imaging techniques. These advancements highlight the potential for improved diagnostic accuracy, treatment planning, and clinical decision-making in medical imaging.

A convolutional neural network (CNN) was developed by Gao et al. to identify acute ischemic stroke patients within a 6-h window for endovascular thrombectomy using computed tomography perfusion and perfusion-weighted imaging. This CNN outperformed support vector machines and random forests, demonstrating its potential for accurate stroke onset time estimation using both CT and MR imaging.

Building on the success of deep learning in stroke diagnosis, another study by Huang et al. utilized deep learning and CT scans to assess lung recovery in COVID-19 Delta variant survivors over 6 months. The findings were promising, with ground-glass opacities disappearing and mild fibrosis in most cases, alongside improved lung prognosis compared to the original COVID-19 strain. In a similar vein, a mixed-effects deep learning model was created by Bridge et al. to diagnose COVID-19 from CT scans, achieving high accuracy and robustness. With an AUROC of 0.930 in external validation, this model outperformed other methods, showcasing potential for clinical application in automated COVID-19 diagnosis.

Transitioning to cardiac imaging, a novel Transformer-ConvNet architecture, MAE-TransRNet, was proposed by Xiao et al. for cardiac MRI registration. This method significantly improved deformable image registration accuracy by combining the strengths of convolutional neural networks (CNN) and Transformers, outperforming state-of-the-art methods on the ACDC dataset.

Extending the application of deep learning to ENT diagnostics, a multi-scale deep learning network, MIB-ANet, was developed by Bi et al. for grading adenoid hypertrophy from nasal endoscopy images. This network outperformed junior E.N.T. clinicians in accuracy and speed, demonstrating its potential for clinical application in automated adenoid hypertrophy grading.

Further advancing medical imaging, an anatomical prior-informed masking strategy for pre-training masked autoencoders was introduced by Wang et al. to enhance brain tumor segmentation. Leveraging brain structure knowledge to guide masking, this method improved efficiency and accuracy on the BraTS21 dataset, outperforming state-of-the-art self-supervised learning techniques. Similarly, a Joint 2D−3D Cross-Pseudo Supervision (JCPS) method was introduced by Zhou et al. for segmenting the carotid vessel wall in black-blood MRI images. This approach, which combines coarse and fine segmentation leveraging both labeled and unlabeled data, significantly enhanced segmentation accuracy, outperforming existing methods.

A systematic review of deep learning techniques for segmenting isointense infant brain tissues in MRI was conducted by Mhlanga and Viriri, analyzing 19 studies from 2012–2022. This review highlighted challenges due to low tissue contrast and overlapping intensity in white and gray matter, with convolutional neural networks (CNNs) being prominently used.

AI-based echocardiographic quantification of global longitudinal strain (GLS) and left ventricular ejection fraction (LVEF) in trastuzumab-treated patients was evaluated by Jiang et al.. They found moderate to strong correlations with conventional methods, suggesting AI's potential as a supplementary tool in clinical settings despite lower feasibility rates. In another study employing echocardiograms, Zhang Y. et al. introduced an automated pipeline that utilizes deep neural networks and ensemble learning to accurately quantify left ventricular ejection fraction (LVEF) and predict heart failure. Their method demonstrated high accuracy and clinical applicability, achieving a Pearson's correlation coefficient of 0.83 with expert analysis and an AUROC of 0.98 for heart failure classification. Furthermore, a semi-supervised contrastive learning network was proposed by Guo et al. for multi-structure echocardiographic segmentation. Evaluated on the CAMUS dataset, it achieved high performance, outperforming existing methods and using fewer parameters. This approach enhances cardiac disease diagnosis and reduces clinician workload.

Finally, for oncology, MRI radiomics-based machine learning models were compared for predicting glioblastoma multiforme prognosis by Zhang D. et al. The DeepSurv model outperformed traditional Cox proportional-hazards and other models, highlighting the potential of deep learning in improving GBM survival predictions.

In conclusion, the integration of data-driven machine learning approaches with prior knowledge marks a significant advancement in medical imaging. The studies reviewed herein underscore the transformative impact of these combined methodologies, offering substantial improvements in diagnostic accuracy, efficiency, and robustness across various medical imaging tasks. This Research Topic significantly contributes to the field by addressing key challenges and paving the way for more reliable and precise medical image analysis, ultimately enhancing patient outcomes and clinical decision-making.

## Author contributions

DZ: Conceptualization, Writing – original draft, Writing – review & editing. JD: Writing – review & editing. CQ: Writing – review & editing. GL: Writing – review & editing.

